# Rehabilitation of Supraspinatus Impingement in a Volleyball Athlete: A Case Report

**DOI:** 10.7759/cureus.51869

**Published:** 2024-01-08

**Authors:** Mansee S Dangare, Saylee S Shedge, Swapnil U Ramteke

**Affiliations:** 1 Sports Physiotherapy, Ravi Nair Physiotherapy College, Datta Meghe Institute of Higher Education and Research, Wardha, IND

**Keywords:** youth athlete, volleyball, physiotherapy rehabilitation, shoulder joint pain, supraspinatus impingement

## Abstract

Volleyball players with supraspinatus tendinopathy commonly present with a spectrum of symptoms, including shoulder pain, especially during the overhead phases of the game. They may experience pain during serves, spikes, or attempting to block at the net. Weakness in the affected shoulder and limited range of motion can impede performance and overall playing experience. Physiotherapy plays a crucial role in managing supraspinatus tendinopathy, focusing on reducing pain, improving shoulder joint range and function, and preventing recurrence. The research question arises as to how the rehabilitation process impacts the recovery and performance outcomes in a volleyball athlete with supraspinatus impingement which is explained as detailed in a case report. The present case is a 21-year-old male volleyball athlete complaining of pain in the anterolateral and posterior aspects of the right shoulder joint and a restricted range of motion while doing abduction and flexion at the shoulder joint for three months. After the orthopedic physical assessment, the patient was diagnosed with supraspinatus tendinopathy. This case report introduces an exact understanding of the rehabilitation tailored specifically to volleyball athletes with supraspinatus impingement.

## Introduction

Supraspinatus tendinopathy is a common source of shoulder discomfort. Tendinopathy is a broad word for a disorder characterized by pain within and surrounding a tendon linked with repeated actions and a reduction in function that occurs after the healing process, unable to repair the tendon effectively [1]. Rotator cuff (RC) tendon ruptures are one of the most prevalent shoulder problems impacting those participating in games and repeated activities connected with work or everyday life [2].

Volleyball is among the most common sports worldwide, with over 200 million athletes participating globally [3]. However, the activity necessitates rapid motions that alter the body's functions and location in the environment, including the horizontal, vertical, and rotating paths. Arm muscles are put under the most strain throughout overhead motions, as they are used in other athletic activities such as basketball, baseball, tennis, and swimming, where RC degenerative conditions are also prevalent [4]. Volleyball is a rapid sport in which heavyweights are delivered via the athlete's shoulder. The spike and jump serve have specific biomechanical characteristics that create shoulder problems. During a given season, 23%-43% of competitive volleyball players report shoulder complaints, whereas 58% of collegiate club-level players have shoulder issues. Despite this, the majority of players continue to practice and compete, with 60% of amateur players having difficulty striking all regions of the court and 60% feeling discomfort while spiking [5]. The five steps of a spike or jump motor movement are method, take off, arm cocking, arm speed, and follow-through. To make the necessary upward connection with the volleyball in indoor volleyball, a player must abduct and rotate their dominant upper extremity at the shoulder and then uncoil it throughout speed. It is necessary to flex, rotate inside the shoulder, and lengthen the elbow when accelerating the upper limb. According to the direction the ball is going, the forearm is pronated to a greater or lesser extent. In indoor volleyball, the roll shot is a tactical off-speed placing shot meant to surprise the opposition. The abilities are broken down into steps for comparing kinematic data providing and spiking: ball contact, speed, and arm cocking [6].

Scapular muscles are vital for stabilizing and regulating the scapula throughout shoulder raising to maintain appropriate posture and kinematics. The initiation of synchronous activation of the scapular muscles is critical for efficient scapular movement throughout shoulder abduction in players participating in overhead games [7]. As they maintain the humeral head centered inside the glenoid cavity, the RC tendons, which serve as dynamic stabilizers of the scapulohumeral joint, are continuously stretched during sports, particularly upward throwing. In the subacromial area, the tendons are constantly moving, which can cause discomfort, thinning, and lesions [8]. When throwing above, there is often more stiffness in the posterior region of the shoulder, which limits internal rotation motions [9]. This may cause the humeral head to shift antero-superiorly, which might result in the occurrence of RC tendonitis. Shoulder tendinopathy is more common in sportsmen who lift weights upward. Interferential therapy (IFT) is a popular therapeutic treatment approach that includes crossing two medium-frequency currents to create a low-frequency pounding action in the tissues beneath the skin. IFT is said to alleviate pain and enhance circulation [10]. 

This study examines the rehabilitation process's critical significance in determining pain relief, increased range of motion of shoulder joints, and performance results of volleyball athletes suffering from supraspinatus impingement, as revealed in a thorough case report. The goal of this study is to provide understanding of the clinical management of supraspinatus impingement, but also practical implications for healthcare professionals and sports practitioners involved in athlete care and rehabilitation.

## Case presentation

Patient information

A 21-year-old, right-hand-dominant male volleyball athlete came to the sports outpatient department (OPD) with the chief complaints of pain in the anterolateral and posterior aspect of his right shoulder joint and difficulty smashing while playing volleyball. He was apparently alright three months back, after which, while playing volleyball, he smashed the ball, and his forearm was blocked in the net. He experienced pain that was sudden in onset and localized to the anterolateral and posterior aspects of the right shoulder joint, but after taking rest, he continued playing matches. The pain had subsided for a couple of months, but while playing in September, the pain again aggravated and was gradual on the onset. He noted that the pain progressively worsened, especially when he performed certain volleyball movements, such as serving and spiking. As the condition worsened, he visited the sports physiotherapy OPD on 20/09/2023 at Ravi Nair Physiotherapy College in Sawangi, where an assessment/evaluation was done. On the visual analog scale (VAS), the score was 7.3/10 on activity and 2.2/10 on rest.

Clinical findings

The patient was conscious and well-oriented. Verbal consent was taken from the patient before performing the physical examination. Vitals were hemodynamically stable. The patient was seen in supine lying. On inspection, it was observed that the patient’s upper limbs were extended and slightly abducted. On palpation, grade 1 tenderness was present on the anterolateral and posterior aspect of the right shoulder joint. Manual muscle testing was examined as given in Table 1 and the right and left shoulder joint range of motion (ROM) was assessed as given in Table 2. A special test of the shoulder joint, i.e. Neer test was positive.

**Table 1:** Manual muscle testing of the right and left shoulder joint Rt: Right; Lt: Left

Muscle	Rt (affected side)	Lt side (non-affected side)
Shoulder flexors	3/5	5/5
Shoulder extensors	2/5	5/5
Shoulder abductors	2/5	5/5
Shoulder adductors	3/5	5/5
Shoulder internal rotators	2/5	5/5
Shoulder external rotators	2/5	5/5

**Table 2:** Range of motion of right and left shoulder joint Rt: Right; Lt: Left; AROM: Active Range of Motion; PROM: Passive Range of Motion

Joint	Rt Active (Affected side)	Rt Passive (Affected side)	Lt Active (unaffected side)	Lt Passive (unaffected side)
Shoulder				
Flexion	0-120^o^	0-125^o^	0-175^o^	0-180^o^
Extension	0-30^o^	0-35^o^	0-55^o^	0-60^o^
Abduction	0-80^o^	0-90^o^	0-175^o^	0-180^o^
Adduction	80^o^-0	90^o^-0	175^ o^ -0	180^o^ - 0
External rotation	0-20^o^	0-25^o^	0-85^o^	0-90^o^
Internal rotation	0-25^o^	0-30^o^	0-65^o^	0-70^ o^

Therapeutic interventions

**Figure 1:**
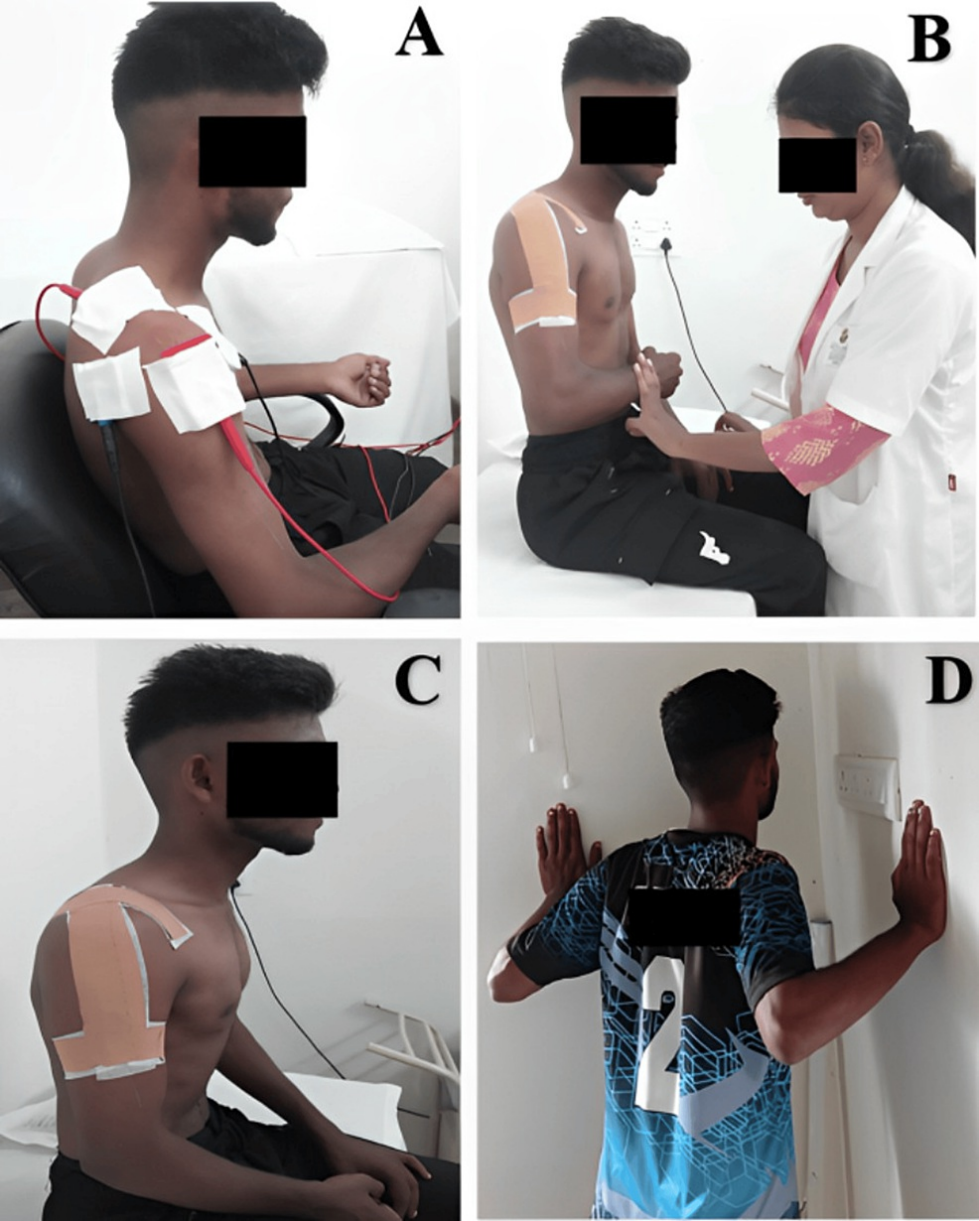
A: Interferential therapy (4 pole vector) for pain relieving; B: Isometrics exercise for external rotation of shoulder joint; C: Inverted J taping to increase the subacromial; D: Wall push-ups in scaption plane

The purpose of physiotherapy and rehabilitation was to decrease pain, improve the range of motion, increase muscle strength, and return to play, as given in Table 3. The patient treated is shown in Figures 1A-1D.

**Table 3:** Goal-oriented physiotherapy protocol IFT: Interferential Therapy; Min: Minutes

Sr.no.	Physiotherapy goals	Therapeutic intervention	Treatment regime/ Dosages
1.	Patient Education	To inform the patient regarding the injury, causes, signs and symptoms.	Teach patients about injury prevention and proper warm-up and cool-down routines.
2.	To reduce pain	Cryotherapy, interferential therapy of alternating medium frequency current over anterolateral and posterior of the right shoulder joint.	Cryotherapy was given for 10-15 mins, IFT (4 pole vector) for 10 mins (for pain relieving).
3.	To improve the strength of muscles	Isometrics exercises of right rotator cuff muscles; wall push-ups in the scaption plane	The patient was instructed to perform two sets of 10 repetitions of active internal and external rotation of the right shoulder joint with maximum resistance over the wrist with three fingers; wall push-ups with 10 repetitions, 2 sets.
4.	Progressive strength training exercises	Initiated with yellow TheraBand exercises internal and external rotation exercises of the right shoulder joint with red TheraBand.	Ten repetitions with two sets initiated with 5-sec hold and then progress to 10 sec holds.
5.	To support anatomical structures and protection from re-injury	Rigid taping over the anterolateral and posterior of the right shoulder joint.	Rigid taping for on day.

Outcome measures

The outcome measures after physiotherapy rehabilitation are given in Tables 4-6.

**Table 4:** Manual muscle testing assessment pre- and post-rehabilitation

Muscle	Pre-rehabilitation	Post-rehabilitation
Shoulder flexors	3/5	5/5
Shoulder extensors	2/5	5/5
Shoulder abductors	2/5	5/5
Shoulder adductors	3/5	5/5
Shoulder internal rotators	2/5	5/5
Shoulder external rotators	2/5	5/5

**Table 5:** Visual analog scale assessment pre- and post-rehabilitation

Scales	Pre-rehabilitation	Post-rehabilitation (After one week)	Post-rehabilitation (After two weeks)
Visual analog scale (on activity- smashing, internal rotation and abduction)	7.3/10	5.1/10	2.1/10

**Table 6:** Range of motion of right shoulder joint (affected side) assessment pre- and post-rehabilitation

Shoulder Joint Movement	Pre-rehabilitation Rt (Affected side)	Post-rehabilitation (After one week)	Post-rehabilitation (After two weeks)
Flexion	0-120^ o^	0-140^ o^	0-170^ o^
Extension	0-30^ o^	0-40^ o^	0-50^ o^
Abduction	0-80^ o^	0-120^ o^	0-170^ o^
Adduction	80^ o^ -0	120^ o^ -0	170^ o^ -0
External rotation	0-20^ o^	0-50^ o^	0-70^ o^
Internal rotation	0-25^ o^	0-45^ o^	0-60^ o^

## Discussion

A study found that shoulder pain and thickened subacromial bursitis (SAB) are common in professional volleyball players [11]. In contrast, there was a noticeable variation in side-to-side problems. This result is in line with other studies on endurance swimmers, who found that swimmers experiencing discomfort for a week after a race had thicker SAB. Cross-sectional studies also support this relationship, with water polo players showing SAB in 63% of players with pain [12]. Elevated SAB thickness in the affected shoulder was seen in non-athletes having unilateral shoulder discomfort throughout upward activities [13]. Effective therapy is nearly responsible for the secondary avoidance of shoulder discomfort. This means promptly providing the indicated athlete with a comprehensive and accurate diagnosis. Following this, they should not engage in any physical activity until they remain asymptomatic and have had a thorough biomechanical examination to make sure that no mal adaptations are hiding beneath the surface that might cause further harm. Currently, less research is available to support the use of shoulder orthoses or shoulder girdle taping to regulate scapular biomechanics in avoiding (or overtime therapy) painful shoulder disorders [14].

Beach volleyball, an Olympic activity that requires frequent upward striking, has been linked to infraspinatus muscle weakness. Nonetheless, knowledge of its incidence, causes, impact on shoulder function, and linkage with shoulder imaging is lacking [15]. Overhead shoulder exercise, excessive use, or trauma cause infraspinatus atrophy, which leads to suprascapular nerve neuropathy. Direct nerve compression at the scapular notch or spinoglenoid notch owing to ganglion cysts or hypertrophied ligaments could lead to this injury [16].

After RC damage, therapeutic techniques such as ice, electrical stimulation, and laser can be used to reduce discomfort, edema, and inflammation while restoring the normal range of motion. Water polo players frequently have shoulder injuries, which include a strange and complicated mix of subacromion deltoid bursitis, RC tendinopathy, and tears, long head biceps tendinopathy, shoulder irritation, and superior labral lesions injuries. The significance of immediate pain relief is shown by studies that demonstrate a 23% drop in electromyographic activity and a 32 percent decrease in external rotation force output in a sore shoulder [17]. Athletes should refrain from engaging in activities including weightlifting or sports-specific exercises like throwing that might reproduce symptoms. In the management of supraspinatus tendinopathy, hyperthermia therapy provided a significant benefit amount in reducing pain and disability when in comparison with ultrasound or pendular swinging and stretching procedures (poor degree of evidence) [18]. Neuromuscular electrical stimulation and isometric training can help alleviate muscle restriction of the RC muscles caused by post-injury discomfort and joint effusion [19]. It has been demonstrated that these methods enhance the creation of external rotation force and encourage muscle activation. A secure and efficient technique to start rehabilitation is with isometric training, which paves the way for more difficult isotonic strengthening exercises [20].

## Conclusions

This case report highlights the efficacy of physiotherapy as a fundamental component in the rehabilitation process known interventions such as targeted exercises and pain management strategies on the recovery trajectory of the volleyball athlete. Supraspinatus tendinopathy is a condition that can significantly impact athletes from various sports. Proper evaluation, diagnosis, and rehabilitation are essential to help athletes return to peak performance while minimizing the risk of recurrent injuries. Physiotherapists play a crucial role in therapeutic rehabilitation for volleyball athletes with supraspinatus tendinopathy. They address pain, optimize shoulder function, correct biomechanical issues, and promote long-term shoulder health, enabling athletes to strongly return to their game and reduce the recurrence risk of injuries. The incorporation of sport-specific biomechanical considerations in the rehabilitation process represents a novel aspect, shedding light on the importance of addressing the unique demands of overhead activities in volleyball.
